# Serum Levels of Lipopolysaccharide and 1,3-*β*-D-Glucan Refer to the Severity in Patients with Crohn's Disease

**DOI:** 10.1155/2015/843089

**Published:** 2015-05-28

**Authors:** Yanmin Guo, Guangxi Zhou, Chong He, Wenjing Yang, Zhenkun He, Zhanju Liu

**Affiliations:** ^1^Department of Gastroenterology, Shanghai Tenth People's Hospital of Tongji University, Shanghai 200072, China; ^2^Department of Infectious Diseases, Huaihe Hospital of Henan University, Kaifeng 475000, China

## Abstract

*Objectives*. Interactions between the host and gut microbial community contribute to the pathogenesis of Crohn's disease (CD). In this study, we aimed to detect lipopolysaccharide (LPS) and 1,3-*β*-D-glucan (BG) in the sera of CD patients and clarify the potential role in the diagnosis and therapeutic approaches. *Materials and Methods*. Serum samples were collected from 46 patients with active CD (A-CD), 22 CD patients at remission stage (R-CD), and 20 healthy controls, and the levels of LPS, BG, and TNF in sera were determined by ELISA. Moreover, sixteen patients with A-CD received anti-TNF monoclonal antibody therapy (infliximab, IFX) at a dose of 5 mg/kg body weight at weeks 0, 2, and 6, and the levels of LPS and BG were also tested at week 12 after the first intravenous infusion. *Results*. Serum levels of LPS and BG were found to be markedly increased in A-CD patients compared with R-CD patients and healthy controls (*P* < 0.05). They were also observed to be positively correlated with CDAI, ESR, and SES-CD, respectively (*P* < 0.05). Furthermore, the levels of TNF in sera had a significant correlation with LPS and BG, respectively. The concentrations of LPS and BG were demonstrated to be significantly downregulated in the sera of A-CD patients 12 weeks after IFX treatment (*P* < 0.05), suggesting that blockade of TNF could inhibit bacterial endotoxin absorption, partially through improving intestinal mucosal barrier. *Conclusions*. Serum levels of LPS and BG are significantly increased in A-CD patients and positively correlated with the severity of the disease. Blockade of intestinal mucosal inflammation with IFX could reduce the levels of LPS and BG in sera. Therefore, this study has shed some light on measurement of serum LPS and BG in the diagnosis and treatment of CD patients.

## 1. Introduction

Crohn's disease (CD), belonging to inflammatory bowel disease (IBD), is one of the most prevalent chronic gastrointestinal diseases in the world, characterized by severe intestinal inflammation of the small and/or large intestine leading to abdominal pain, recurrent diarrhea, perianal fistula, and clinical manifestations of bowel obstruction [1]. Increasing data have suggested that CD is a multifactorial disorder, resulting from the interplay among abnormal immunoregulation, genetics, intestinal mucosal barrier dysfunction, and intestinal flora disequilibrium [2–4]. Evidence has demonstrated that T helper (Th) cells are involved in the production of chronic intestinal inflammation and that Th1- and Th17-mediated immune responses are dominant in the gut mucosa during the progress of CD [5].

Gut flora, which can coexist with the host throughout the digestive tract in variable concentrations (the upper limit is 10^11^–10^12^/g of luminal contents in the colon), is referred to as the largest microbial reservoir in the body [6]. This community plays the vital roles in the host, ranging from helping various enzymes processing indigestible materials to regulating the immune system and constituting intestinal mucosal barrier [7]. An external “anatomical” barrier and an inner “functional” immunological barrier consist of gut mucosal barrier. The former constitutes a large amount of coexisted gut flora, the mucous layer, and the intestinal epithelial monolayer, while the latter is comprised of a complex network of immune cells and gut-associated lymphoid tissues, which is organized in a specialized and compartmentalized system [8]. These components together sustain the maintenance of the delicate balance of intestinal homeostasis and gut permeability. Once the balance is disrupted, the bacterial infection and inflammation occur, which ultimately can aggravate the development of CD.

Experimental data and clinical observation have demonstrated an important role of the complex intestinal microflora in the development of mucosal inflammation [1]. Compared with healthy controls, ulcerative colitis (UC) patients present greater bacterial density on the colonic mucus layer [7]. And there is a higher abundance of invasive* E. coli* in mucosal biopsy specimens, especially in ileal specimens, from patients with CD compared with healthy subjects [9]. Adherent-invasive* E. coli* cause great damage to the host, through invading epithelial cells, replicating within macrophages, and then promoting the formation of granuloma in vitro [10, 11]. The decreased diversity of intestinal microflora is related to the gut mucosal inflammation in patients with CD. Relevant study has shown that the diversity of microflora in CD is reduced to 50% compared with controls and to 30% in UC [12]. Another treatment for IBD called fecal microbiota transplantation has been confirmed to be effective, which is reported to restore the new balance of intestinal microbiota by clustering normal intestinal flora to the patients, which are extracted from healthy donors [13]. Mesalamine, as an anti-inflammatory drug routinely used in IBD patients, could decrease inflammation of intestinal mucosa characterized by a decrease of* Escherichia/Shigella *in the gut [14, 15]. Furthermore, some promising data have illustrated that the use of antibiotics in IBD could induce remission of the inflammation or prevent inflammatory relapse. After receiving ileal pouch-anal anastomosis surgery, UC patients may develop ileal mucosal inflammation and colonic bacteria can be tested in the ileal pouch, where faecal stasis occurs [12]. Relevant animal experiments also suggest that no inflammation occurs in the gut mucosa on aseptic conditions, while inflammation occurs after transplantation of gut microbiota, and the severity of inflammation is dependent on the bacterial strains that existed in gnotobiotic animals [16]. Taken together, these data have proven that intestinal microbiota play an important role in the occurrence and development of intestinal inflammation.

Our recent study has confirmed that serum bacterial toxins derived from* C. difficile*,* Salmonella*,* S. aureus*, and* E. coli* O157, respectively, could be detected in serum samples of active IBD patients, and the levels were correlated with the severity and treatment of the disease. Levels of bacterial toxins are decreased in CD patients after receiving anti-TNF mAb (infliximab, IFX) treatment by improving intestinal inflammation, suggesting that the bacterial toxins in sera could help us evaluate the progression of IBD [17]. To further determine the association between serum toxins and CD, we detected the levels of LPS and BG in the sera of CD patients and analyzed the correlation with clinical features. The results demonstrated that the serum levels of LPS and BG were found to be significantly increased in active CD patients and correlated with ESR, TNF, and the severity of disease. Furthermore, IFX treatment also decreased the levels of LPS and BG in the sera. Therefore, serum levels of LPS and BG could be used as the index to predict the progress and severity of CD.

## 2. Materials and Methods

### 2.1. Subjects

This study included 46 patients with active-CD (A-CD) (28 male, 18 female; aged 12–53 years old), 22 CD patients at remission stage (R-CD) (17 male, 5 female; aged 16–47 years old), and 20 healthy controls (8 male, 12 female; aged 24–44 years old) from August 2013 to October 2014 at the Department of Gastroenterology, the Shanghai Tenth People's Hospital of Tongji University (Shanghai, China). The disease durations were all more than 3 months. Diagnoses of CD were established by conventional clinical, radiological, endoscopic, and histological criteria [18]. The severity of disease and intestinal mucosal lesions were graded by the Crohn's disease activity index (CDAI) and simple endoscopic score for CD (SES-CD). A-CD and R-CD were defined by SES-CD >2 and ≤2 [19, 20], respectively. The behaviors of CD patients were assessed according to the Montreal classification system [21]. Clinical data of CD patients regarding age, gender, classification, treatment (e.g., 5-ASA, corticosteroids, azathioprine, and IFX), and smoking history were collected. Written informed consent was obtained from each participant before experiments. The study was approved by the Ethics Committee of Shanghai Tenth People's Hospital. All research involving human subjects was undertaken conforming to the provisions of the latest revision of World Medical Association's Declaration of Helsinki.

### 2.2. Analysis of LPS and BG

Blood samples were drawn from A-CD patients, R-CD patients, and healthy controls after an overnight fast. Sera were collected via centrifugation and used up within 10 minutes. The levels of LPS and BG were measured using photometric detection test kits (Beijing Jinshan Science and Technology Co., Ltd., Beijing, China). The results were analyzed and a cut-off point was set at 10 pg/mL according to the manufacturer's instruction.

### 2.3. Analysis of TNF by ELISA

Three milliliters of peripheral blood was drawn from 46 A-CD and 22 R-CD patients after overnight fast. Sera were extracted after centrifugation and stored at −80°C freezer until use. Each serum sample was tested for TNF by enzyme-linked immunosorbent assay (ELISA) according to the manufacturer's instructions and the ELISA kit was purchased from eBioscience (San Diego, CA, USA). The sensitivity of each assay was 10 pg/mL.

### 2.4. Detection of LPS and BG before and after IFX Treatment

Sixteen A-CD patients (12 male, 4 female; aged 13–37 years old) were all naive to biological agent therapy and received anti-TNF mAb (IFX; Cilag AG, Schaffhausen, Switzerland) treatment at a dose of 5 mg/kg body weight at weeks 0, 2, and 6 [22]. It was administered by a 2 h intravenous infusion. Serum samples were collected at weeks 0 and 12, after the first IFX therapy, and analyzed for LPS and BG according to previous method.

### 2.5. Statistical Analysis

Data were expressed as mean ± standard deviation (SD). The levels of serum LPS and BG were expressed as the log_10_ transformation. Statistical analysis was performed using SPSS version 20.0 (SPSS, Inc., IBM Corporation, Somers, NY). Simple analyses were conducted by the one-way analysis of variance or the nonparametric alternative, the Kruskal-Wallis test, to compare continuous variables. Pearson correlation was used for studying the correlation of LPS, CDAI, SES-CD, ESR, CRP, and TNF, as well as BG. The results were interpreted at the significance level of 0.05, variables with *P* < 0.05 were reported as statistically significant, and *P* < 0.01 was considered obviously statistically significant.

## 3. Results

Table 1 demonstrates the baseline demographics of CD patients' age, sex, classification, smoking history, medical treatment, disease duration, and the concentrations of LPS and BG in the sera. The average age at the time of A-CD diagnosis was 33.07 ± 15.70 years (28 male and 18 female), with 6 patients being A1, 28 patients being A2, and 12 patients belonging to A3. The scores of CDAI were 194.40 ± 65.71 (range from 39.60 to 335.80). The scores of SES-CD were 9.84 ± 5.59 (range from 2.40 to 23.40). The values of ESR were 34.49 ± 18.37 mm/h (range from 2.0 to 64 mm/h). The values of CRP were 42.04 ± 41.42 mg/L (range from 3.27 to 157 mg/L). The concentrations of LPS were 44.41 ± 89.44 pg/mL, being 1.19 ± 0.59 by log_10_ formation (range 0.30 to 2.70 pg/mL), and the concentrations of BG were 306.88 ± 373.82 pg/mL, being 2.03 ± 0.74 by log_10_ formation (range 0.7 to 3.00 pg/mL).

**Figure 1:**
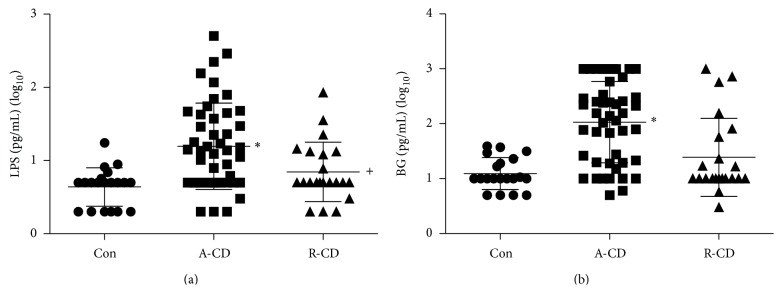
Serum levels of LPS and BG (log_10_ formation) in A-CD patients. (a) Levels of LPS were significantly increased in patients with A-CD as compared with R-CD and healthy controls (Con) (^+^ *P* < 0.05, A-CD VS R-CD; ^*^ *P* < 0.01, A-CD VS Con); (b) Serum levels of BG were markedly increased in patients with A-CD as compared with R-CD and Con (^*^ *P* < 0.01, A-CD VS R-CD or Con).

As shown in Figure 1, the levels of LPS and BG were found to be markedly increased in A-CD patients compared with those R-CD patients and healthy controls (*P* < 0.05). There was no significant difference between R-CD patients and healthy controls (*P* > 0.05).

**Figure 2:**
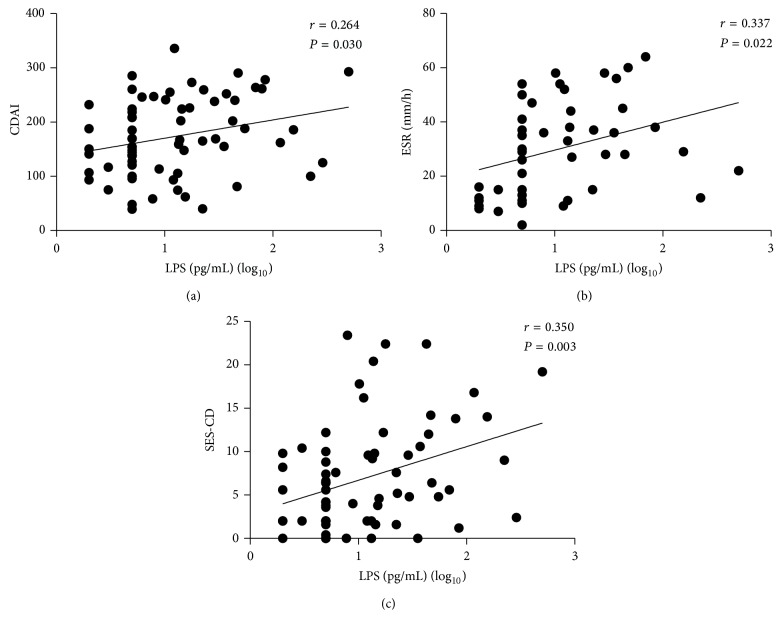
Correlation analysis among CDAI, ESR, and SES-CD and the levels of LPS in CD patients. Correlation between CDAI and LPS ((a), *r* = 0.264, *P* = 0.03) in all CD patients. Correlation between ESR and LPS ((b), *r* = 0.337, *P* = 0.022) in all CD patients. Correlation between SES-CD and LPS ((c), *r* = 0.350, *P* = 0.003) in all CD patients.

**Figure 3:**
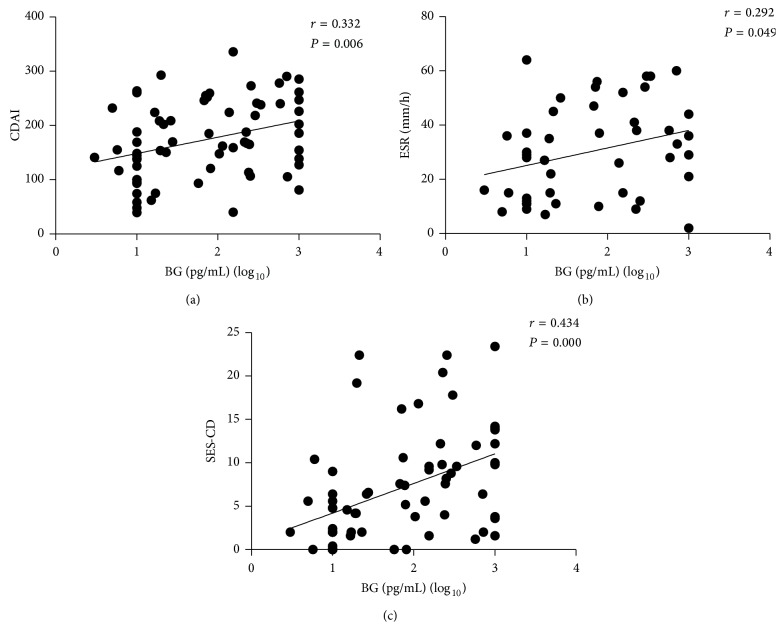
Correlation analysis among CDAI, ESR, and SES-CD and the levels of BG in CD patients. Correlation between CDAI and BG ((a), *r* = 0.332, *P* = 0.006) in all CD patients. Correlation between ESR and BG ((b), *r* = 0.292, *P* = 0.049) in all CD patients. Correlation between SES-CD and BG ((c), *r* = 0.434, *P* = 0.000) in all patients.

To determine the relationship between the levels of LPS and CDAI, ESR, SES-CD, and CRP and the relationship between the levels of BG and CDAI, ESR, SES-CD, and CRP, we did Pearson correlation analysis. As shown in Figure 2(a), the levels of LPS and CDAI showed a weaker association with Pearson correlation coefficient (*r* = 0.264, *P* < 0.05). Moreover, weaker concordances were also observed between the levels of LPS and ESR (*r* = 0.337, *P* < 0.05) and SES-CD (*r* = 0.350, *P* < 0.01), respectively (Figures 2(b) and 2(c)).  Figure 3(a) demonstrates that the levels of BG and CDAI had a weaker association with Pearson correlation coefficient (*r* = 0.332, *P* < 0.01). Similarly, weaker concordances were also observed between the levels of BG and ESR (*r* = 0.292, *P* < 0.05) and SES-CD (*r* = 0.434, *P* < 0.01), respectively (Figures 3(b) and 3(c)). Unexpectedly, no significant difference was seen between CRP and the levels of LPS (*r* = 0.169, *P* = 0.172) and the levels of BG (*r* = 0.182, *P* = 0.141), respectively.

**Figure 4:**
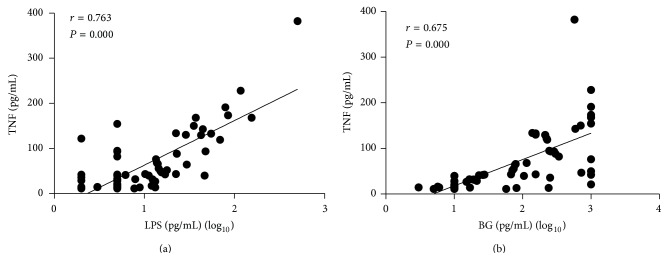
Correlation between TNF and LPS or BG in CD patients. The levels of TNF were analyzed in 46 A-CD and 22 R-CD patients by ELISA. The levels of TNF were significantly associated with the concentration of LPS ((a), *r* = 0.763, *P* = 0.000) or BG ((b), *r* = 0.675, *P* = 0.000), respectively.

We also analyzed the concordance relationship of the levels of LPS or BG between the levels of TNF, showing that the levels of TNF in sera were significantly correlated with LPS (*r* = 0.763, *P* = 0.000) and BG (*r* = 0.675, *P* = 0.000), respectively (Figures 4(a) and 4(b)).

**Figure 5:**
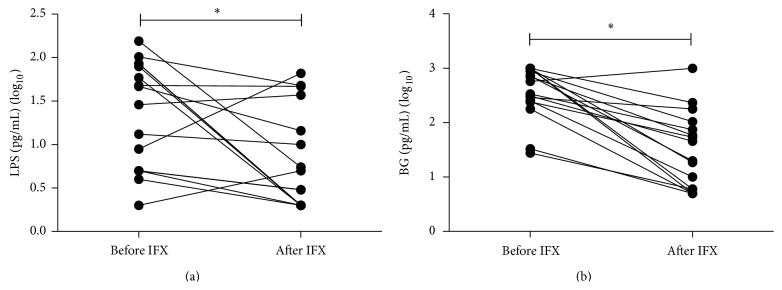
The levels of LPS and BG are decreased with IFX. Sixteen A-CD patients received IFX at a dose of 5 mg/kg body weight at weeks 1, 2, and 6. The sera were collected 12 weeks after IFX therapy and analyzed for LPS and BG. (a) The levels of LPS were significantly decreased (^*^ *P* < 0.05) as compared with those before IFX treatment. (b) The levels of BG were significantly decreased (^*^ *P* < 0.01) as compared with those before IFX treatment.

Moreover, 16 A-CD patients received IFX treatment at a dose of 5 mg/kg body weight at weeks 0, 2, and 6. Sera were then collected 12 weeks after the first IFX therapy and analyzed for LPS and BG. As shown in Figures 5(a) and 5(b), the levels of LPS and BG were significantly decreased (*P* < 0.05 and *P* < 0.01) as compared with those before IFX treatment.

## 4. Discussion

Microbiota coexist with the human body and maintain a delicate balance under normal condition. In the gastrointestinal tract, there are greater densities of bacterial species, which are also known as intestinal flora, and the common bacterial divisions are* Firmicutes, Bacteroidetes, Actinobacteria, Proteobacteria,* and* Verrucomicrobia* [23, 24]. To date, increasing data have reported massive diversity of bacterial species and* Bacteroidetes* and* Firmicutes* are referred to be the dominant bacterial groups in the gut [25]. Recently, evidence has demonstrated that gut microbiota are involved in the pathogenesis and etiology of IBD, reinforcing the view that interactions between intestinal microbes and the mucosal immune system promote the progression of IBD [26, 27]. Under abnormal conditions,* C. difficile* colonizes in the gut mucosa and interacts with intestinal epithelial cells, which induces an inflammatory cascade and then results in intestinal diseases such as diarrhea and pseudomembranous colitis [28]. Therefore, flora disequilibrium, if not properly treated, may be unfavorable to the host, leading to intestinal inflammation, mucosal damage, infection of opportunistic pathogen, and maybe some severe diseases, such as IBD [29]. However, little is known about toxin in the sera of IBD patients, especially the bacterial toxin LPS and fungal toxin BG. In this study, we found that the levels of LPS and BG were markedly increased in A-CD patients compared with R-CD and healthy controls, being significant association between CDAI, ESR, SES-CD and TNF. Importantly, IFX therapy induced the reduction of sera levels of LPS and BG.

LPS, one important component in outer membrane of Gram-negative bacteria, plays a critical role and is composed of three main parts including lipid A, O-side chain, and core oligose [30]. Apart from maintaining structural integrity, LPS, when released into the blood stream, also could induce inflammatory responses, which further result in various diseases, such as CD [31]. Breakdown of the gut mucosal barrier during chronic CD progression allows translocation of bacterial products such as LPS from the gut into the circulation. In DSS colitis, increased levels of LPS in sera can be detected, as a result of the damaged inner mucus layer, disrupted mucus barrier, and activated immune cells, which are all induced by the translocation of commensal bacterial [32].

Toll-like receptors (TLRs), known as pattern recognition receptors expressed by various cells in the gastrointestinal tract, can recognize a wide range of microbial fragments, especially antigens derived from the microbiota [33]. Generally, LPS is specifically sensed by the innate immune system via TLR4 and functions to induce cytokine responses to whole Gram-negative bacteria. At normal circumstances, because of the low expression of TLR4, intestinal epithelial cells are unresponsive to TLR stimuli and therefore no inflammation occurs. However, under inflammatory conditions, TLR4 is highly increased markedly in intestinal epithelial cells, which further exacerbate the inflammatory response. Related reports have demonstrated that TLR4 is expressed in the murine intestinal mucosa, and similar to human CD, its expression is upregulated in the inflamed colon [34]. Therefore, increased expression of epithelial TLR4 is associated with the gut inflammation in CD. TLR4 activation in intestinal epithelial cells is also known to regulate the expression of tight junction proteins. Activation of TLR4 impairs tight junctions, increases intestinal permeability, and then results in bacterial translocation [33]. Therefore, the serum level of LPS was found to be increased obviously in active CD patients and positively correlated with the inflammatory severity.

Previous studies have shown that no inflammation occurred in germ-free mice; however, after colonization of transient adherent-invasive* E. coli*, chronic inflammation occurred and the severity of inflammation was correlated with levels of bioactive LPS, which provided strong evidence that LPS from Gram-negative bacteria can trigger severe inflammation [35, 36].* E. coli* is the dominant strain in early and chronic ileal lesions of CD and mainly abnormally adheres to intestinal epithelial cells. In vitro, most* E. coli* strains can be isolated from the ileal mucosa of CD patients. Our previous study has demonstrated that active IBD patients had significant high positive rates of* E. coli* toxin. In our study, we have detected high levels of LPS in CD patients which were also highly associated with severity of disease, which further confirmed the proinflammatory effect of LPS.

In CD patients, increased intestinal epithelial permeability precedes clinical relapse by as much as 1 year, suggesting that a permeability defect may exacerbate the progression of disease [37]. The increased permeability of the intestinal epithelium prompted the absorption of microorganisms in the gut (e.g., LPS and BG), T cells, B cells, and macrophages and dendritic cells in intestinal mucosal epithelial tissue are then activated and a large number of inflammatory mediators appeared. Therefore, abnormal intestinal barrier function may be involved in the pathogenesis of CD. Evidence has shown that inflammatory severity of colitis attenuated and serum LPS levels decreased in colitic mice, after being treated with Gelatin tannate (a material can form a protective film over gut barrier), suggesting that LPS entered the circulation via abnormal gut barrier permeability [38]. Another study has demonstrated that ileitis model mice manifested higher survival rates and lower levels of LPS compared with untreated mice after receiving treatment of LPS antagonist polymyxin B, which further confirmed proinflammatory effect of LPS [39]. Therefore, serum LPS could be used as a target for the amelioration of inflammation.

BG is a common and major component of all fungal cell walls and plays an important role in the inflammatory response to the organism. Previous study has found that the levels of BG are elevated in CD patients and provided direct evidence of fungal infection in CD patients [40]. Our study demonstrates that BG is detectable in the peripheral blood CD patients without known active fungal infection and is significantly higher in A-CD patients, which could represent translocation of fungus components into the bloodstream. BG could recognize the C-type lectin-like *β*-glucan receptor dectin-1, which is mainly expressed in the surface of macrophages, then activate macrophages to release proinflammatory cytokines (such as IL-12 and TNF), and finally prompt the development of acute inflammation [41]. Additionally, it can also stimulate arachidonic acid to release COX2, which further exacerbates the progress of inflammation. These two pathways are both regulated via the TLR2 dependent pattern [41]. In our results, we also found that BG levels are associated with TNF, ESR, and the severity of disease activity.

It is generally accepted that CRP and CDAI are preferred in CD because of well correlation with disease activity in clinical practice. Moreover, SES-CD is a more intuitive assessment of colonic mucosal lesions and reflects real state of disease [20, 42]. Therefore, we adopt SES-CD to define the state of disease in our study. Although CDAI and SES-CD are widely used to evaluate the disease activity of CD, the strong subjectivity has demonstrated that these scores do not represent real state of disease [43–45]. Those factors may lead to weaker correlation between LPS or BG and CDAI, ESR, and SES-CD.

It is now widely accepted that TNF is a vital inflammatory mediator in the pathogenesis of CD by involving in different inflammatory pathways and the subsequent downstream responses including NF-*κ*B activation, cytotoxicity, and induction of proinflammatory cytokines and chemokines, as well as antiapoptotic effects, which are triggered by the combination of TNF receptors and its ligands [46]. Mononuclear cells in peripheral blood of CD patients produce high levels of TNF compared to healthy controls and the concentrations of TNF are consistent with laboratory parameters of disease. Moreover, our study also proved that the serum LPS and BG levels are significantly correlated with the serum levels of TNF, suggesting that LPS and BG may play a proinflammatory role through the induction of TNF.

Currently, most of the efficient biologic therapies developed so far in CD aim at neutralizing the proinflammatory activity. IFX, a fully human monoclonal IgG anti-TNF antibody, could specifically decrease the serum TNF levels in CD patients, which further alleviate the intestinal inflammation and promote to restore the gut epithelial barrier [47] and demonstrated beneficial activity in induction and maintenance of clinical remission, mucosal healing, and reduction in surgeries and hospitalizations. In our research, IFX treatment could reduce the serum levels of LPS and BG by improving the intestinal inflammation.

In summary, serum levels of LPS and BG are significantly increased in CD patients and positively correlated with the severity of the disease. Blockade of intestinal mucosal inflammation with IFX could reduce the levels of LPS and BG in sera. Therefore, this work has shed some light on measurement of serum LPS and BG in the diagnosis and treatment of CD patients.

## Figures and Tables

**Table 1:** Demographic characteristics and clinical features of the CD patients.

	A-CD (*n* = 46)	R-CD (*n* = 22)
Age at diagnosis		
A1 (≤16 years)	6	1
A2 (17–40 years)	28	14
A3 (>40 years)	12	7
Gender (male/female)	28/18	17/5
Classification		
L1	3	8
L2	8	2
L3	35	12
Current smoking	2	3
Current treatment		
5-ASA	14	5
Azathioprine	4	4
Corticosteroids	12	1
IFX	16	12
Age at CD diagnosis (year)	33.07 ± 15.70 (12–53)	35.95 ± 10.65 (16–47)
Duration (month)	36.15 ± 40.14 (0.75–168)	30.14 ± 25.61 (2.0–96)
LPS (pg/mL) (log_10_⁡)	1.19 ± 0.59 (0.30–2.70)	0.84 ± 0.41 (0.30–1.93)
BG (pg/mL) (log_10_⁡)	2.03 ± 0.74 (0.7–3.00)	1.39 ± 0.71 (0.48–3.00)
ESR (mm/h)	34.39 ± 18.37 (2.0–64)	21.53 ± 2.97 (7–38)
CRP (mg/L)	42.04 ± 41.42 (3.27–157)	31.06 ± 57.20 (0.5–203)
CDAI	194.40 ± 65.71 (39.6–335.80)	128.36 ± 62.13 (40.20–278.1)
SES-CD	9.84 ± 5.59 (2.40–23.40)	1.11 ± 0.93 (0.00–2.00)

5-ASA: 5-aminosalicylate; SD: standard deviation; M/F: male/female; CRP: C-reactive protein; CDAI: CD activity index; SES-CD: simple endoscopic score for CD; TNF: tumor necrosis factor; LPS: lipopolysaccharide; BG: 1,3-*β*-D-glucan.
